# Neurotransmitter and neuropeptide regulation of mast cell function: a systematic review

**DOI:** 10.1186/s12974-020-02029-3

**Published:** 2020-11-25

**Authors:** Huaping Xu, Xiaoyun Shi, Xin Li, Jiexin Zou, Chunyan Zhou, Wenfeng Liu, Huming Shao, Hongbing Chen, Linbo Shi

**Affiliations:** 1 Department of Rehabilitation, The First Affiliated Hospital of Nanchang University, Nanchang, 330006 Jiangxi Province China; 2 Department of Anesthesiology, The First Affiliated Hospital of Nanchang University, Nanchang, 330006 China; 3 School of Food Science, Nanchang University, Nanchang, 330047 Jiangxi Province China; 4 Department of Pathogen Biology and Immunology, School of Basic Medical Sciences, Nanchang University, 461 Bayi Avenue, Nanchang, 330006 Jiangxi Province People’s Republic of China; 5 State Key Laboratory of Food Science and Technology, Nanchang University, Nanchang, 330047 Jiangxi Province China

**Keywords:** Neuron-mast cell interaction, Neurotransmitters, Neuropeptides

## Abstract

The existence of the neural control of mast cell functions has long been proposed. Mast cells (MCs) are localized in association with the peripheral nervous system (PNS) and the brain, where they are closely aligned, anatomically and functionally, with neurons and neuronal processes throughout the body. They express receptors for and are regulated by various neurotransmitters, neuropeptides, and other neuromodulators. Consequently, modulation provided by these neurotransmitters and neuromodulators allows neural control of MC functions and involvement in the pathogenesis of mast cell–related disease states. Recently, the roles of individual neurotransmitters and neuropeptides in regulating mast cell actions have been investigated extensively. This review offers a systematic review of recent advances in our understanding of the contributions of neurotransmitters and neuropeptides to mast cell activation and the pathological implications of this regulation on mast cell–related disease states, though the full extent to which such control influences health and disease is still unclear, and a complete understanding of the mechanisms underlying the control is lacking. Future validation of animal and in vitro models also is needed, which incorporates the integration of microenvironment-specific influences and the complex, multifaceted cross-talk between mast cells and various neural signals. Moreover, new biological agents directed against neurotransmitter receptors on mast cells that can be used for therapeutic intervention need to be more specific, which will reduce their ability to support inflammatory responses and enhance their potential roles in protecting against mast cell–related pathogenesis.

## Background

Both the nervous and immune systems play critical roles in regulating processes required to protect against external threats, respond to acute stressors, and maintain physiological homeostasis [1]. As “first responders” of the immune system, MCs are omnipresent in the body. They are localized in association with PNS and the brain, where they are closely aligned, anatomically and functionally, with neurons and neuronal processes throughout the body. MC numbers are particularly high in tissues innervated by small-caliber sensory A-delta and C-fibers, which are responsible for pain transmission; the close anatomic associations between MCs and nerves are especially evident at sites of inflammation [2, 3]. Thus, the existence of the neural control of MC functions has long been proposed [4].

MCs are found near externally exposed surfaces such as the epithelium of the skin, the gastrointestinal tract mucosa, meningeal membranes of the central nervous system (CNS), and lung airways [5]. This proximity to the external environment makes MCs prime candidates to mount rapid responses to external stimuli and the internal microenvironment, thus, contributing to many biological and pathological processes. Besides their well-known role in allergic inflammation, MCs have diverse physiological roles, including innate defense against infections, modulation of adaptive immunity, angiogenesis, and tissue homeostasis. Moreover, MCs also exist in different regions of the human heart, including the coronary arteries, myocardium, aorta, and adipose tissue. Hence, mediators from activated MCs can contribute directly to the pathogenesis of cardiometabolic diseases and their associated complications [6].

**Fig. 1:**
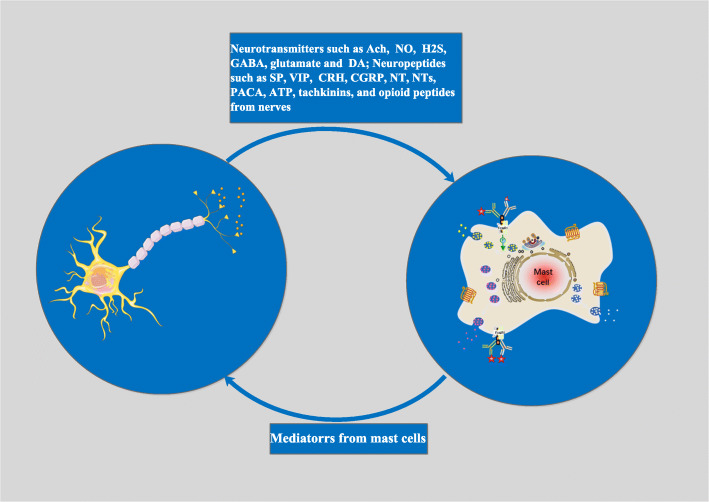
The mast cell–nerve interaction: a paradigm of neuroimmune crosstalk. MCs express receptors for various neurotransmitters and neuropeptides and are modulated by them, allowing neural control of mast cell function. Conversely, mast cells also communicate with nerves by secreting mediators. Ach, acetylcholine; NO, nitric oxide; H_2_S, hydrogen sulfide; GABA, gamma-aminobutyric acid; DA: dopamine; SP, substance P; VIP, vasoactive intestinal peptide; CRH, corticotrophin-releasing hormone; CGRP, calcitonin gene-related peptide; NTs, neurotrophins; NT, neurotensin; PACAP, pituitary adenylate cyclase–activating polypeptide; ATP, adenosine triphosphate. This figure contains adapted images obtained from Servier Medical Art by Servier

As a neuroimmune archetype, MCs participate as an essential intermediary between the immune and nervous systems, exhibiting a range of functions in both systems. MCs express receptors for various classical neurotransmitters, including acetylcholine (Ach), the gaseous neurotransmitters nitric oxide (NO), hydrogen sulfide (H_2_S), gamma-aminobutyric acid (GABA), dopamine (DA), and glutamate; neuropeptides such as substance P (SP), vasoactive intestinal peptide (VIP), corticotropin-releasing factor (CRF), calcitonin gene-related peptide (CGRP), neurotrophins (NTs) and neurotensin (NT), adenosine triphosphate (ATP), tachykinin, opioid peptides, and other neuromodulators (Fig. 1). Consequently, modulation provided by these neurotransmitters and neuromodulators allows neural control of MC functions and gets involved in the pathogenesis of mast cell–related disease states. These diverse neurotransmitters and neuropeptides produce different effects on MC functions executed through their receptors in MCs and range from direct activation to enhancement or repression of responses or even no responses to other stimuli [1]. The roles of individual neurotransmitters and neuropeptides in regulating MC action have been reported in healthy and disease states. However, a systematic review concerning neurotransmitter and neuropeptide regulation on MCs is lacking. Here, we review the recent advances in our understanding of the contribution of neurotransmitters and neuropeptides to mast cell activation and the pathological implications for mast cell–related disease states.

## Neurotransmitter regulation of mast cells

### Acetylcholine regulation of mast cells

The structural relationship of vagal afferents and mast cells was examined, and the data support that the vagus may have some influence over MC function. The most recent advances in understanding the immunoregulatory role of the vagal nerve have focused on the cholinergic anti-inflammatory pathway [7]. In this pathway, acetylcholine (Ach), the principal cholinergic neurotransmitter that usually exerts its action through binding to nicotinic cholinergic receptors (nAChR), stimulates macrophages to inhibit the production of pro-inflammatory cytokines via binding to the alpha7 nicotinic acetylcholine receptor (α7 nAChR) found on macrophages. The homomeric α7 receptor is one of the most abundant nAChRs in the nervous system, and it also is present on various non-neuronal cells, including MCs [8]. This receptor plays an essential role in cognition, memory, pain, neuroprotection, and inflammation, suggesting it is a potential target for those pathological processes [9].

Several studies have demonstrated that the α7 nAChR-dependent anti-inflammatory pathway attenuates specific MC responses, which might explain the reported therapeutic effects of its agonists on diverse models of allergic diseases. Agonists of nicotinic receptors, including the α7-specific agonist, GTS-21, inhibited immunoglobulin E (IgE)-induced degranulation of mucosal-type murine bone marrow–derived MCs in a dose-dependent manner, which was prevented by α7 antagonists [10]. Tumor necrosis factor (TNF) production from lipopolysaccharide (LPS) treatment of MCs was suppressed by α7 nAChR agonists through targeting extracellular regulated protein kinases 1/2 and TNF-α converting enzyme activation, suggesting that inflammatory mediators produced by MCs are negatively controlled by nicotinic receptor activation [11]. The production of interleukin-6 (IL-6) and NO by peripheral blood mononuclear cells that are elevated in chronic obstructive pulmonary disease (COPD) patients and associated with impaired lung function, also was inhibited by α7 nAChR agonists, supporting the therapeutic potential of α7 nAChR agonists in COPD [12]. On the other hand, the non-specific nAChR agonist, nicotine, inhibited IgE-induced cysteinyl leukotrienes and cytokine production without affecting rat mast cell/basophil cell line (RBL-2H3) MC degranulation through alpha 7/alpha 9/alpha 10-nicotinic receptors [13]. Exposure to nicotine decreased the allergen-specific IgE levels and potentiated Treg cell proliferation against the allergen in BALB/c mice [14]. Additionally, some studies have identified mucosal MCs as early targets of the nutrition-induced vagal anti-inflammatory reflex during acute inflammation. Administration of lipid-rich nutrition inhibited the release of rat MC protease II through activation of the cholinergic anti-inflammatory pathway, following induction of MC-mediated postoperative ileus [15, 16].

On the other hand, some evidence suggests the occurrence of cholinergic activation of MCs. Vagal stimulation has been shown to increase the histamine content of intestinal mucosal MCs, without their apparent degranulation, and these changes were prevented by subdiaphragmatic vagotomy [17]. The non-specific nAChR agonists, Ach, choline, and nicotine, induced mast cell degranulation, in vitro, without leukotriene B4 or TNF-α secretion, and nicotinic acetylcholine receptor antagonists blocked cholinergic stimulation [18]. Host expression of the α7 nAChR is required in mice for epicutaneous 4-(methylnitrosamino)-1-(3-pyridyl)-1-butanone (NNK, an important component of thirdhand smoke) exposure to exacerbate cockroach antigen–induced asthma pathology through MCs [8]. Eosinophilia induced by house dust mites was positively modulated by α7 nAChRs, although eosinophilia induced by ovalbumin (OVA) was negatively modulated by α7 [19]. Interestingly, some studies failed to show any effect of acetylcholine on rat MC degranulation. Nicotine treatment did not reduce β-hexosaminidase levels in bronchoalveolar lavage fluid, but it strongly reduced lung T helper 2 cell responses in antigen-challenged Norway rats [20].

These contradictory observations indicate that the acetylcholine receptor regulation of MCs is complex. The functional outcome of vagal input might be affected by the acetylcholine receptor subtype and the presence of allergen-specific and cell-specific differences [7, 19]. Furthermore, neuropeptides, such as VIP and NO, are released from cholinergic and inhibitory non-adrenergic, non-cholinergic neurons, which also modulate MC activity, thus, affecting the outcome of vagal stimulation [21].

### NO regulation of mast cells

The gaseous neurotransmitter nitric oxide (NO), is co-stored and released with Ach, in addition to VIP [7]. NO is endogenously produced in the body by three isoforms of NO synthase (NOS), neuronal NOS (nNOS, NOS 1), endothelial NOS (eNOS, NOS3), and inducible NOS (iNOS, NOS 2). Numerous studies have been conducted on the relationship between NO and MC mediator release. Most studies have demonstrated that both endogenous and exogenous NO inhibit MC degranulation. NO donors such as sodium nitroprusside (SNP) inhibited the release of histamine from isolated rat peritoneal MCs that is evoked by compound 48/80 or the calcium ionophore, A23187 [22–24]. The NO donors also dramatically decreased IgE-induced MC degranulation and mRNA expression of IL-4, IL-6, and TNF from rat MCs [25, 26]. SNP, and other NO donors such as spermine-NO, and SIN1, significantly reduced MC degranulation in the mesentery after ischemia and reperfusion in rats [27]. On the other hand, NOS inhibitors can reverse the inhibitory effects of NO on MC function. Pretreatment of enriched rat peritoneal MCs with the NOS inhibitor *N*^*G*^-nitro-l-arginine methyl ester, (l-NAME) markedly enhanced *E. coli* LPS-evoked histamine release [28]. Inhibition of NO synthesis increased epithelial permeability associated with increased release of rat MC protease II [29]. Some studies have investigated the molecular mechanisms underlying NO regulation of MC function. NO effectively downregulates human MC adhesion, which might be attributed to inhibition of the cysteine protease, calpain, an enzyme that is associated with the control of integrin activation in other cell types; calpain inhibition is most likely mediated through nitrosylation of the thiol group at its active site [30]. Other studies have attempted to detect the direct role of neural-derived NO on MCs. NO generated by intestinal nNOS mediated the anti-inflammatory effects of intestinal ischemic preconditioning (IPC) associated with reduced MC degranulation in an IPC model [31]. However, vagal stimulation has been shown to protect against injury-associated increases in intestinal permeability [32, 33]. Therefore, the degree of involvement of nitrergic parasympathetic nerves and MCs has not been resolved.

Notably, NO and NOS are important regulators of migraine, as indicated by experimental, neuropathological, biochemical, and pharmacological data. The role of NO-mediated dural MC degranulation in migraine pathogenesis has been hypothesized in many studies [34, 35]. In vivo application of the nitric oxide donor, glyceryl trinitrate (GTN), led to a striking increase in MC degranulation via an as yet unknown mechanism [35]. This effect was completely blocked by inhibition of endogenous NO production, although the direct application of an exogenous NO donor on dural MCs did not cause their degranulation ex vivo [35]. Thymoquinone pretreatment prevented the activation of meningeal MCs, and their numbers were not increased by exposure to GTN in in vivo migraine rats [36]. These observations indicate a role for NO-mediated MC degranulation in migraine [36]. It has been suggested that targeting NO production with nNOS inhibitors might be an excellent opportunity for selective NOS inhibition in migraine treatment, as it is strongly associated with migraine pathophysiology [37]. Also, acute administration of L-NAME, a non-specific inhibitor of NO synthase attenuated the anti-allergic effects of sumatriptan, a 5-hydroxytryptamine 1B/1D (5HT1B/1D) agonist, indicating the involvement of inducible NOS in allergic inflammation [38]. When considering the numerous roles of MCs in many biological and pathological processes, the importance of NO and NOS system regulation of MC function cannot be underestimated. We foresee that both basic and clinical research in this area will continue for decades to come.

### H_2_S regulation of mast cells

H_2_S was identified as the third gasotransmitter in 1996 following the discoveries of the biological importance of NO and carbon monoxide (CO) [39]. It plays a physiological role in a range of functions, including synaptic transmission, vascular tone, angiogenesis, inflammation, and cell signaling [40]. It has been reported that H_2_S inhibits mast cell actions and modulates many pathophysiological processes, including inflammation and allergic reactions. H_2_S might protect the heart during heart failure by suppressing local renin levels through inhibition of MC infiltration and renin degranulation [41]. H_2_S inhibited antigen-induced degranulation in RBL-2H3 cells in vitro, and inhalation of the hydrogen sulfide donor, NaHS, reduced OVA-induced airway hyper-reactivity and MC degranulation, although it did not affect MC counts or plasma IgE levels [42]. The novel H_2_S donor, 4-carboxy-phenyl isothiocyanate PhNCS-COOH, prevented the increase in [Ca^2+^](i) elicited by Ca^2+^ ionophores and Fc epsilon RI (FcεRI) activation of MCs, and it also attenuated the phosphorylation of spleen tyrosine kinase, cytosolic phospholipase A2, and phosphodiesterase gamma 1 in antigen-stimulated RBL-2H3 cells [43]. These data suggest that H_2_S donors could be exploited as complementary therapeutic approaches in H_2_S-related pathogenesis.

### Gamma-aminobutyric acid and mast cells

Gamma-aminobutyric acid (GABA) is a well-known inhibitory neurotransmitter in the mammalian CNS. Its beneficial role in preventing and treating various diseases in the CNS and non-neuronal peripheral tissues and organs has been documented widely. Some reports link GABA to the inhibition of MC activation in allergies. GABA suppresses degranulation in rat basophilic leukemia RBL-2H3 cells via the GABA(B) receptor on the cell surface [44, 45]. Moreover, the synthetic analog of GABA, gabapentin, significantly reduced dextran-induced mouse paw edema, which depends on MC activation [46]. The administration of GABA in a dose-dependent manner reduced the development of AD-like skin lesions in mice by suppressing serum IgE and splenocyte IL-4 production [47]. In the IL-4/Luc/CNS-1 Tg mouse model of atopic dermatitis (AD), the use of fermented soybean products containing a high concentration of GABA decreased ear thickness, dermis thickness, and infiltrating MCs [48]. Therefore, the anti-allergic properties of GABA have been used to ameliorate allergic symptoms such as AD.

### Glutamate and mast cells

Glutamate is well known as a major excitatory mediator of the nervous system and non-neuronal cells [49]. It acts by binding to various glutamate receptors, and glutamate receptor signaling has been implicated in various pain conditions, including tendinopathy [50, 51]. Some studies have reported that MCs located within healing tendons express glutamate receptors, raising the possibility that MCs may respond to glutamate. Glutamate receptor, NMDAR1, was profoundly upregulated in a rat model of tendon rupture, and tendon injury was accompanied by extensive MC degranulation, whereas MCs in uninjured tendons showed low or nondetectable glutamate receptor expression [52]. Glutamate not only induced MC degranulation and the upregulation of glutamate receptors in MCs at both the mRNA and protein levels but also upregulated gene expression, including transcription factors, such as Egr2, Egr3, and, in particular, FosB, and pro-inflammatory components such as IL-6 and CCL2 in MCs from injured tendon tissues in rats [53]. Together, these observations establish glutamate as an effector of MC function. Further studies are required to evaluate whether the glutamate-glutamate receptor axis in MCs can be exploited for therapeutic purposes in neurogenic inflammation and the inflammatory reaction that accompanies tendon healing [54]. Also, transdermal administration of high-molecular-weight (HMW, 1100 kDa) poly-gamma glutamate (PGA) microneedles (MNs) significantly reduced clinical dermatitis scores, epidermal thickness, and MC infiltration in mice by downregulating IgE, suggesting that gamma-PGA MNs represent an innovative, safe, and reliable therapeutic strategy for AD management [55].

### Dopamine and mast cells

As a component of the brain’s dopamine system, dopamine (DA) is an essential neurotransmitter in the CNS and regulates locomotion, emotion, cognition, and neuroendocrine secretion via binding to its specific receptors [56]. Several studies have reported relationships between DA and MC activation. However, some results are controversial. For instance, the D1-like dopamine receptor antagonist, SCH 23390, significantly reduced the passive cutaneous anaphylaxis reaction (PCA), a classic model of IgE/mast cell–mediated allergic skin reaction, in mice and DA enhanced bone marrow-derived MC degranulation, which was abrogated by SCH 23390 [56]. These results suggest that DA promotes IgE/mast cell–mediated allergic reactions. On the other hand, administration of the DA precursor levodopa (l-DOPA), which is converted to DA in the brain, with benserazide ameliorated PCA in mice, suggesting that dopamine antagonizes allergy [57, 58]. It also has been demonstrated that the toxic effects of methamphetamine (METH) depend on the similarity of its chemical structure to DA and the dopamine D3 receptor (D3R) plays an important role in METH-mediated neurotoxic effects [59]. METH administration suppresses LPS-induced MC activation in D3R^+/+^, but not D3R^−/−^ mouse intestine, and D3R also was involved in METH-mediated modulation of LPS-induced expression of TLR4 and downstream mitogen-activated protein kinase and nuclear factor kappa-B signaling molecules in mouse bone marrow-derived mast cells [59]. Clinical evidence indicates a causal relationship between rheumatoid arthritis (RA) and the dopaminergic system [60]. D3R on MCs also may be involved in superoxide dismutase (SOD)-mediated pathogenesis of RA, and it may inhibit SOD production by negatively modulating TLR4 signaling on MCs in RA patients [61].

## Neuropeptide regulation of mast cells

### Substance P regulation of mast cells

Substance P (SP), a sensory neuropeptide released at the peripheral ends of sensory nerves during inflammation, is perhaps the best-known and most studied neurotransmitter related to MC activation. It has been reported that SP stimulates both degranulation and chemokine production of MCs, which might be involved in the pathogenesis of various neuro-inflammatory diseases such as psoriasis, multiple sclerosis, asthma, rhinitis, AD, rheumatoid arthritis, atherosclerosis, and others. Moreover, SP-mediated MC activation also contributes to the pathogenesis of cardiometabolic diseases, including atherosclerosis, abdominal aortic aneurysm, obesity, and diabetes mellitus, as well as complications associated with these diseases [6]. In addition to triggering MC activation, SP can lower the threshold for MC activation in response to subsequent stimuli [7]. It also primes toll-like receptor (TLR) 2-mediated activation of human MCs by upregulating TLR expression and potentiating signaling pathways associated with TLR, suggesting that neuronal responses may influence innate host defense responses [62]. Also, SP induces expression of functional corticotropin-releasing hormone receptor-1 (CRHR-1), while CRH induces neurokinin-1 (NK-1) gene expression in LAD2 human leukemic MCs, thus potentiating inflammatory diseases affected by stress [63].

**Fig. 2:**
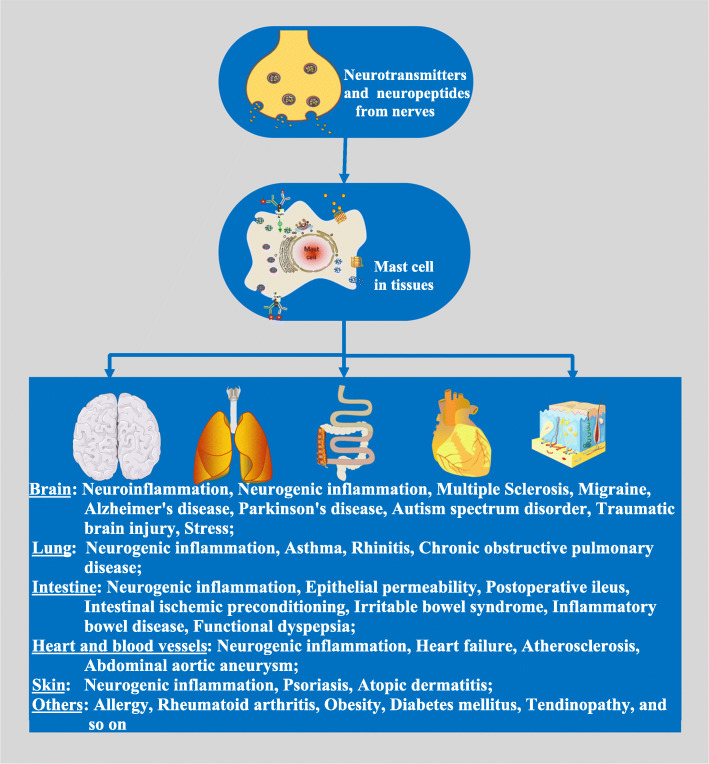
Neural regulation of mast cells. Neurotransmitters such as Ach, NO, H2S, GABA, glutamate, and DA as well as neuropeptides, including SP, VIP, CRF, CGRP, NTs, NT, PACAP, ATP, opioid peptides, and other neuromodulators, modulate the function of mast cells in the meningeal membranes of the CNS, epithelial lining of the skin, airways of the lung, mucosa of the gastrointestinal tract, and so on, contributing to a diverse array of physiological and pathologic functions. This figure contains adapted images obtained from Servier Medical Art by Servier

The neural release of SP and SP binding to its receptors on the MC surface is one mechanism of MC activation (Fig. 2). Many previous experimental studies have demonstrated that SP can induce MC activation in a NK-1 receptor (NK-1R)-dependent fashion [6]. Current evidence also suggests that SP and NK-1R are viable targets for interventions that specifically address various dermatologic conditions, and a selective NK1 receptor antagonist might be a viable treatment option for patients with psoriatic pruritus [64, 65]. SP/NK-1R is essential to cause increased numbers of mature MCs in the hypertensive heart, although NK-1R is not required for the activation of cardiac MCs in vivo [66]. However, mounting evidence currently suggests that SP activates MCs primarily through the G protein–coupled, Mas-related gene X2 receptor (MrgprX2) [67, 68]. SP might signal via MRGPRX2 to activate human cultured MCs, resulting in a type of MC degranulation characterized by the rapid release of small spherical granules [69]. On the other hand, naturally occurring missense MRGPRX2 variants display a loss of function phenotype for MC degranulation in response to SP, indicating that individuals expressing certain naturally occurring MrgprX2 missense variants might have a degree of protection against neurogenic inflammation [70, 71]. In vitro SP activation of human MCs led to the release of multiple pro-inflammatory cytokines and chemokines via MRGPRX2, independent of NK-1R, and MRGPRX2 mediated neurogenic inflammation and pain in an incision mouse model [72]. In the allergic contact dermatitis (ACD) mouse model, MC activation via Mrgprb2 evoked non-histaminergic itching in mice, independent of the IgE-FcεRI-histamine axis [73]. The MRGPRX2-targeting antagonist DNA, aptamer-X35, reduced histamine release and anaphylactic reactions in a rat anaphylaxis model [74]. Therefore, SP action on MCs via human MrgprX2 may explain the lack of clinical efficacy of NK1 receptor antagonists in disorders associated with neurogenic inflammation [75]. Also, the mouse Mrgprb2 and human MRGPRX2 in MCs recognize Quorum-sensing molecules (QSMs) secreted by bacteria, which results in MCs eliciting antibacterial mediator release, suggesting MRGPRX2 is a potential therapeutic target for controlling bacterial infections [76].

Nevertheless, MRGPRX2-mediated MC activation might be more complicated than is currently understood. Other small, endogenous basic peptides such as VIP, cationic peptide drugs, and derivatives of the common MC activator compound, 48/80, also activate mouse MCs via MRGPRB2, the orthologue of human MRGPRX2 [67, 77]. Similarly, MRGPRX2 antagonists in humans might inhibit SP-mediated receptor activation and MRGPRX2 activation by other ligands [78]. Therefore, the MRGPRX2 receptor is thought to be involved in most of the peptide stimulus-related activation of human MCs in various physiologic and pathologic conditions [79]. It is suggested that assessing MRGPRX2 activation might prove valuable as a rapid, high-throughput, economic, mechanism-based screening tool for early MC degranulation identification during preclinical safety evaluations of peptide-based therapeutics [80]. This is important because MRGPRX2-mediated MC degranulation is an important potential safety issue for peptide-based therapeutics [80]. However, some studies suggest that the identification of mast cell MRGPRX2 receptor targets for therapeutic intervention needs to be more specific, although many studies indicate that antagonists of MRGPRX2 might possess therapeutic benefits in those conditions. MRGPRX2 is generally more sensitive to SP than the mouse orthologue, MrgprB2, indicating that Mrg receptors may underlie species-specific differences in MC responses to SP [69]. MRGPRB3 expressed on rat peritoneal MCs can respond to MRGPRX2 ligands and regulate MC-mediated activities, suggesting that MRGPRB3 might be the MRGPRX2 orthologue in rats [81, 82]. Moreover, the expression and function of MRGPRX2 receptors on primary human MCs isolated from different anatomic sites are heterogeneous, which might result in heterogeneous de novo synthesized mediators that are released due to the MRGPRX2-mediated MC activation [83].

### VIP regulation of mast cells

Vasoactive intestinal peptide (VIP), a 28 amino acid peptide, exerts its action on cells via two G protein–coupled receptors, VPAC1 and VPAC2 [84]. VIP is widely expressed in the nervous, endocrine, and immune systems [85]. It is considered a true neuroimmunoendocrine mediator that modulates innate and adaptive immunity and exhibits a predominantly anti-inflammatory action [85]. Mast cells also express VIP receptors allowing VIP to have the potential to modulate MC mediator secretion. Furthermore, there is evidence that VIP does protect diverse tissues by suppressing MC mediator secretion. VIP can act as a neuroprotective agent to support neuronal survival by modulating MC behavior in physiological and pathological conditions, such as neurological diseases. Systemically administered VIP can attenuate motor response changes, neuronal cell death, and myelin loss, characteristic in a rat model of Parkinson’s disease [86]. The protective effect of VIP in this model could, at least in part, be mediated by brain MCs, as VIP treatment changes the ultrastructural morphology of MCs in a manner that is characteristic of “piecemeal” degranulation [86]. VIP decreased the number of MCs on both sides of the dura mater in sympathectomized rats [87]. In Parkinsonian rats, VIP was thought to preserve neurons by inducing native brain MCs to adopt a non-degranulating phenotype that could secrete numerous neuroprotective substances, such as nerve growth factor (NGF) and heparin [88]. This evidence provides an understanding of how VIP might be applied to manage neurodegenerative diseases [89]. VIP substantially protected liver tissue from septic shock in rats and testicular tissue from detorsion injury via inhibiting MC activity [90, 91]. The presence of numerous MCs with decreased degranulation is strongly associated with VIP-positive nerve fibers in the papillary dermis of aged skin, suggesting that VIP may influence MC distribution and function in aged skin [92].

However, many current studies have implicated a role for VIP in increased intestinal permeability via VIP-receptors (VPAC1/VPAC2) on MCs, proposing that MCs and VIP act as regulators of the intestinal barrier and inflammation [93]. Compared to healthy individuals, irritable bowel syndrome (IBS) patients expressed higher levels of VIP and tryptase, increased numbers of MCs, a higher percentage of MCs that expressed VPAC1, and increased bacterial passage, which was significantly diminished after blocking with anti-VPACs and ketotifen, demonstrating that mechanisms of increased translocation included MCs and VIP [94]. Upregulation of intestinal mucosal MCs expressing VPAC1 in close proximity to VIP in inflammatory bowel disease (IBD) and murine colitis, suggests that communication between MCs and VIP is upregulated during IBD and mouse colitis [95]. In a functional dyspepsia (FD) mouse model, both VIP receptor antagonists and ketotifen significantly reversed the proton pump inhibitor-mediated enhanced intestinal permeability and decreased transepithelial electrical resistance in the duodenum and jejunum, implicating VIP and MCs in the underlying mechanisms of FD [96].

VIP also modulates immunological reactions in airways. Pretreatment of MCs with VIP in the human airway increases the ability of smooth muscle cell-derived fractalkine (FKN) to attract MCs in asthma [97]. Current evidence suggests that the expression of VIP specific receptors, including VPAC2 and chemoattractant receptor–homologous molecule expressed on Th2 cells (CRTH2) receptor on MCs and basophils, provides an opportunity for VIP to act on these cells and promote allergic disease pathogenesis [98]. Thus, the mechanisms underlying the different effects of VIP are unknown but are likely related to localized VIP concentrations, the differential expression of classical VIP receptor subtypes, and micro-environmental factors [1].

### Corticotropin-releasing factor regulation of mast cells

Corticotropin-releasing hormone (CRH), also known as corticotropin-releasing factor (CRF), is a 41 amino acid peptide released from hypothalamic neurons. The CRF system, including a family of peptide-related CRFs and the related family of urocortins (I–III), is a major stress regulatory system in the body [99]. CRF mediates its action through G protein–coupled receptors (GPCRs), corticotropin-releasing factor receptor subtype 1 (CRF1), and corticotropin-releasing factor receptor subtype 2 (CRF2). The role of the CRF system has been extensively studied in the CNS regarding hypothalamic–pituitary–adrenal axis (HPA axis) regulation and neurobehavioral paradigms. It also is highly active in peripheral immunological and infectious challenge conditions [100–102].

Studies have examined the role of CRF receptor signaling in MC degranulation responses to immunologic and psychologic stressors. It is known that CRF receptor activation increased MC function in neurological diseases. In stress and neuroinflammatory conditions, elevated CRF increases vascular permeability and activates glia and inflammatory cells, including MCs, to release additional inflammatory mediators [103, 104]. Patients with centralized pain syndromes often present with hyper- or hypocortisolism and altered downstream signaling from the HPA axis, including increased MC infiltration and activation, which can lead to sensitization of nearby nociceptive afferents [105]. Stress can worsen a broad range of inflammation-associated diseases such as AD and autism spectrum disorder (ASD) via stimulation of MCs by CRH and other neuropeptides, leading to increased vascular permeability and inflammation [106]. Previous studies also have demonstrated that CRF interacts with its receptors on subepithelial MCs, indicating that MCs and CRF might regulate the intestinal barrier and inflammation [107]. CRF1, acting as a critical modulator of MC degranulation and stress-induced pathophysiology activation, enhances systemic MC degranulation, which promotes gastrointestinal leakage and systemic anaphylaxis in mice [108]. Activation of the CRF signaling pathway enhances MC degranulation, which is associated with visceral hypersensitivity, and promotes gastrointestinal leakage in functional dyspepsia and IBS [109, 110].

Furthermore, studies have investigated the molecular mechanisms underlying CRF regulation of MC function. Although CRF1 activation did not directly induce MC degranulation, it enhanced Ca^2+^ release from intracellular stores, which potentiated the degranulation responses triggered by diverse MC stimuli [108]. However, MC CRF2 is a negative global modulator of stimulus-induced MC degranulation and limits the severity of IgE-mediated anaphylaxis and stress-related disease pathogenesis [111]. Moreover, there are unique interactions between NT and CRF to increase MC-dependent skin vascular permeability in rodents, and it may contribute to autoimmune and inflammatory diseases that become worse with stress [112]. We believe that exploring the connections between stress and MCs is essential to clarify the pathogenesis and develop effective treatments for diseases that become worse with stress and involve inflammation.

### CGRP regulation of mast cells

Calcitonin gene-related peptide (CGRP) is a 37 amino acid neuropeptide neurotransmitter, and its receptors are found in all organs, but especially in sensory neurons. The effects of CGRP on the cardiovascular system have been studied intensively previously. CGRP regulates cardiac excitability, microvascular permeability, vascular smooth muscle tone, and angiogenesis [113]. CGRP also is highly expressed in the central terminals of the trigeminal nerve and the trigeminal ganglion (TG), where CGRP is often co-released with SP. It is a potent vasodilator that is released during neurogenic inflammation and contributes to the pathology of migraine; anti-CGRP treatment has been proposed as a target for primary headache therapies, including migraine [114]. In contrast, CGRP is protective in models of inflammatory bowel disease and hypertension and is a critical neuropeptide involved in the development and modulation of auditory responses [115–118].

CGRP acts through the CGRP receptor, which is a heterodimer consisting of the calcitonin receptor-like receptor (CLR) and the type 1 receptor activity–modifying protein (RAMP1)–Gs complex, as determined by Volta phase-plate cryo-electron microscopy [119]. However, CGRP can activate multiple receptors and could have more than one endogenous receptor. The recent identification of the CGRP-responsive calcitonin receptor/RAMP1 complex (CTR/RAMP1), (AMY1 receptor-amylin subtype 1 receptor) in the trigeminovascular system warrants deeper consideration of the molecular identity of CGRP receptor(s) involved in the pathophysiology and potential treatment of migraine [120, 121].

There are reports linking mast cell activation to CGRP in headache pain. Studies in rodent models suggest that the potential involvement of CGRP and the ensuing activation of meningeal MCs and resident immune cells can activate the headache pain pathway [122, 123]. Salmon calcitonin alleviated migraine-like pain by modulating CGRP release at different levels, including the generation and conduction sites of migraine pain and MC behavior in the dura mater of rats [124]. However, some studies have reported that CGRP had no direct modulatory effects on meningeal MC activation. In the GTN-induced headache rat model, GTN infusion did not cause receptor-mediated MC degranulation through the release of CGRP, as the CGRP antagonist, olcegepant, had no effect on GTN-induced MC degranulation in vivo [35]. However, the density of CGRP-containing nerve fibers increased in the dura mater as did CGRP release, suggesting the CGRP receptor mechanism is involved in GTN-induced MC activation [34, 125].

Ongoing activation of meningeal MCs is not mediated by peripheral CGRP signaling and does not contribute to the development of mCHI-evoked cephalic mechanical pain hypersensitivity in a mild closed head injury (mCHI) male rat model [126]. However, the presence of granule-containing MCs is required for the development of latent mechanical sensitization [126]. In vitro CGRP generates direct peritoneal MC degranulation, but with reduced chemical messengers [127]. Thus, the role of direct modulatory effects on MC activation by CGRP needs further investigation.

### Neurotrophin regulation of mast cells

Neurotrophins (NTs), comprise a broad family of biomolecules, including NGF, brain-derived neurotrophic factor (BDNF), neurotrophin-3 (NT-3), and neurotrophin-4/5 (NT-4/5), which support the growth, survival, and differentiation of developing and mature neurons. Studies indicate a primary role for NTs in the development, survival, and degranulation of mast cells [128]. NGF is the prototypical and best-characterized neurotrophin. Its concentrations are elevated in numerous inflammatory and autoimmune states in conjunction with increased MCs [129]. NGF also exerts its proinflammatory action on MCs and may be involved in the development of brain diseases and related disorders [130]. However, several other recent reports demonstrate that NGF does not affect MC action. NGF treatment in vitro does not lead to increased levels of TNF-α in bone marrow-derived MCs, and qRT-PCR data indicate that MCs express negligible levels of NGF receptors, thereby explaining their lack of response to NGF [131]. Mouse MCs are not essential to heat hypersensitivity induced by NGF [132].

### Neurotensin regulation of mast cells

Neurotensin (NT) is another neuropeptide that is secreted locally under stress. It induces degranulation and VEGF release from human mast cells, which can be enhanced by CRH-induced neurotensin receptor (NTR) expression on MCs and blocked by the NTR antagonist, SR48692 [112]. Moreover, the mutual interaction between neurotensin and CRH in MCs may contribute to allergy symptoms that worsen with stress, as seen in ASD [133]. However, NT reduced mouse MC protease and malondialdehyde in lung homogenates in a murine hapten-induced asthma model, and the NTR1 antagonist did not reverse the NT-mediated reduced mouse MC activation [134]. Moreover, NT-based chimeric peptide and endomorphin-2 also reduced the concentration of mouse MC protease in a similar model [135].

### Pituitary adenylate cyclase–activating polypeptide regulation of mast cells

Pituitary adenylate cyclase–activating polypeptide (PACAP) has a possible role in the pathophysiology of primary headaches through stimulation of cAMP formation in anterior pituitary cells. Mast cell degranulation also is involved in PACAP-induced migraine. PACAP-38 induces marked vasodilation and degranulation of dural MCs and has a much stronger degranulation effect on rat peritoneal and dural MCs than VIP and PACAP-27, but MC degranulation is not mediated via the known PACAP1–38 receptors [136, 137]. It is suggested that PACAP-38 and PACAP (6-38) degranulate rat meningeal MCs via a putative new PACAP-receptor, the Orphan MrgB(3)-Receptor [138].

### Adenosine triphosphate regulation of mast cells

ATP is known to act as a neurotransmitter and might be a key molecule responsible for the vicious cycle between meningeal mast cells and the nervous system. Extracellular ATP exhibits its effects through the two primary types of purinergic receptors, ionotropic P2X (P2X1-7) and metabotropic P2Y (P2Y1-14) [109]. ATP and the converted product, adenosine, induce synergistic MC degranulation through P1 and P2 receptor co-activation in an ecto-nucleotidase-enriched environment [139]. It was demonstrated that ATP and BzATP (a P2X agonist) increased calcium in human MCs and induced MC degranulation by activating P2X7 receptors [140–142]. The P2X4 receptor plays an essential role in ATP-induced upregulation of MC degranulation in response to Ag, and also promotes the Ag-induced systemic and intradermal passive anaphylaxis responses in vivo [143]. Moreover, activation of the P2X4 receptor (P2X4R) is involved in the synergistic effect of prostaglandin (PG) E2 and ATP on murine MC degranulation [144]. According to the purinergic hypothesis of migraine, the powerful algogen extracellular ATP is a key mediator of this disease [145]. Extracellular ATP activates trigeminal neurons and meningeal MC degranulation via P2X7 receptors and activates nociceptive firing in meningeal trigeminal afferents via the amplified degranulation of resident MCs resulting in direct excitatory action on the nerve terminals, which aggravates the migraine pain [146, 147]. These results indicate that the P2X receptor might be a potential therapeutic target for allergic diseases and migraine.

### Tachykinin regulation of mast cells

The tachykinin family member, hemokinin-1 (HK-1), can induce mast cell degranulation and contribute to the development of experimental asthma in mice via the NK-1R on murine MCs [71, 148–150]. However, the tachykinin, Neurokinin A (NKA), acting alone did not affect either MC granule or cytokine release, but it downregulated the intensity of IgE-initiated MC activation [151]. Intradermal administration of NKA inhibited MC inflammatory functions in the skin via the NK2R, indicating that NKA/NK2R signaling is anti-inflammatory in a murine contact hypersensitivity model [151].

### Opioid peptides and mast cells

The main role of endogenous opioid peptides is pain modulation. They mediate their analgesic effects by acting on opioid receptors that are distributed widely in the CNS as well as other tissues and organs, including the immune system, indicating that opioids exert effects in the periphery [152]. Synthetic opioids, a treatment limited by associated adverse effects, are a mainstay in managing moderate to severe pain and remain the most effective analgesics currently available [153]. It is well known that a range of therapeutically useful opioids degranulate MCs. Most reactions to opioids are non-IgE-mediated and cause direct MC degranulation and histamine release at clinically used doses [154]. Intradermal injection of morphine, hydromorphone, methadone, or morphine metabolites, morphine-6-glucuronide and morphine-3-glucuronide, resulted in a significant cromolyn-sensitive canine skin flare after injection [155]. Exposure of human MCs to morphine and hydromorphone or morphine metabolites, morphine-3-glucuronide and morphine-6-glucuronide, resulted in a robust increase in MC degranulation, but these effects were not mediated through a classical opioid receptor [155].

Intrathecal infusion of morphine that activated Mas-related G protein–coupled receptor degranulated MCs, activated fibroblasts, and resulted in intrathecal mass formation [156]. The activity of DMT-DALDA (H-Dmt-D-Arg-Phe-Lys-NH2; Dmt = 2′,6′-dimethyltyrosine), a selective mu opioid agonist, was equivalent to morphine in producing MC degranulation, although it was greater than a 1000-fold more potent in producing analgesia, suggesting a possible lower risk in producing a spinal mass at equivalent analgesic concentrations [157]. Moreover, the Dmt (2′,6′-dimethyl-L-Tyr) moiety of DMT-DALDA was found to represent the driving force for the high potency and agonist activity at the mu opioid receptor (MOR), and the key amino acid residues Y148^3.33^ and Y326^7.42^ were responsible for DMT-DALDA binding to MOR [158]. On the other hand, opioids can induce IgE-mediated MC degranulation. A few cases of opioid allergy confirmed with opioid drug provocation testing, the gold standard for the diagnosis of IgE-mediated allergy, have been reported [159], but the risk for newly suspected IgE-mediated reactions caused by opioids is low in patients [160]. Also, several opioid compounds, including morphine, can activate the human LAD2 MC line and human skin MCs through MRGPRX2 [161, 162]. However, morphine was an incomplete secretagogue because it did not induce the de novo synthesis of arachidonic acid metabolites from human MCs [162]. Therefore, it is likely that opioids stimulate MC degranulation contributing to neurogenic inflammation, which is central to both pain and pruritus, concomitant to its analgesic effect in the CNS [163]. Thus, targeting MCs may improve opioid analgesia in patients with pain conditions in which MCs are activated to resolve the unmet clinical problem of distressing itch/pain and reduce opioid requirements for pain management [163].

## Conclusions

We have briefly reviewed potential neurotransmitters and neuropeptides that modulate mast cell actions, focusing on recent publications. Their regulation of MCs is implicated in many pathophysiological processes, suggesting that neural regulation contributes to health and disease and presents a range of potential therapeutic applications (Fig. 2). The existing literature provides a complex picture of MC actions that might be a fundamental component or protective in a diverse array of physiological and pathologic functions. The different outcomes of MC activation might exhibit significant genetic variation among individuals and represent additional factors that need to be considered. Given the inherent heterogeneity and plasticity of MCs, the local tissue microenvironment, cell subtype, and neurotransmitter and neuropeptide receptor subtype likely determine the outcome of MC degranulation. On the other hand, MCs also directly trigger neuron activation through mediators, including cytokines, histamine, neurotransmitters, and neurotrophic factors, causing acute activation and long-lasting changes in excitability and neuronal phenotypes. Hence, the full extent to which such interactions influence health and disease is still unclear, and a complete understanding of the mechanisms underlying the relationship between MCs and the nervous system is lacking.

A more thorough understanding of MC-nerve interactions will likely provide valuable insights into how the immune and nervous systems coordinate multiple aspects of homeostatic control and could suggest areas to target the MC-nerve functional unit to provide improved therapeutic effects in both immune and neurological disorders [1]. Perhaps the ability of MCs to interact with the nervous system indicates their importance in diseases where excessive neuronal activity is present. Future considerations include the validation of animal and in vitro models, which incorporates the integration of microenvironment-specific influences and the complex, multifaceted cross-talk between MCs and various neural signals. Moreover, the identification of MC receptor targets for therapeutic intervention needs to be context-specific. New biological agents directed against neurotransmitter receptors on MCs for therapeutic intervention also need to be more specific. In particular, the ability of MCs to support inflammatory responses needs to be reduced, and their potential protective roles in MC-related pathogenesis need to be enhanced. Neurotransmitters and neuropeptides also affect FcεRI-mediated MC activation, which is the critical mechanism in type I hypersensitivity. For example, stimulation of the P2X4 receptor (P2X4R) enhanced FcεRI-mediated degranulation, but α7 nAChR, NO donors, H_2_S, CRF2, and NK2R stimulation negatively modulated degranulation. These results indicate that the neural control of type I hypersensitivity by neurotransmitters and neuropeptides acts through their receptors on MCs. Hence targeting their receptors may be a novel strategy for controlling FcεRI-mediated allergic reactions.

## Data Availability

Not applicable.

## Abbreviations

MC(s): Mast cell(s)
PNS: Peripheral nervous system
CNS: Central nervous system
Ach: Acetylcholine
NO: Nitric oxide
H_2_S: Hydrogen sulfide
GABA: Gamma-aminobutyric acid
DA: Dopamine
SP: Substance P
VIP: Vasoactive intestinal peptide
CRH: Corticotrophin-releasing hormone
CGRP: Calcitonin gene-related peptide
NTs: Neurotrophins
NT: Neurotensin
PACAP: Pituitary adenylate cyclase–activating polypeptide
ATP: Adenosine triphosphate
α7 nAChR: Alpha7 nicotinic acetylcholine receptor
IgE: Immunoglobulin E
TNF: Tumor necrosis factor
LPS: Lipopolysaccharide
IL-6: Interleukin-6
COPD: Chronic obstructive pulmonary disease
RBL-2H3: Rat mast cell/basophil cell line RBL-2H3
OVA: Ovalbumin
SNP: Sodium nitroprusside
l-NAME: *N*^*G*^-nitro-l-arginine methyl ester
IPC: Intestinal ischemic preconditioning
GTN: Glyceryltrinitrate
FcεRI: Fc epsilon RI
AD: Atopic dermatitis
PGA: Poly-gamma glutamate
MNs: Microneedles
PCA: Passive cutaneous anaphylaxis reaction
METH: Methamphetamine
D3R: Dopamine D3 receptor
RA: Rheumatoid arthritis
SOD: Superoxide dismutase
TLR: Toll-like receptor
NGF: Nerve growth factor
IBS: Irritable bowel syndrome
IBD: Inflammatory bowel disease
FD: Functional dyspepsia
CRF1: Corticotropin-releasing factor receptor subtype 1
CRF2: Corticotropin-releasing factor receptor subtype 2
ASD: Autism spectrum disorder
MOR: Mu opioid receptor
